# Deep learning‐based dose prediction for stereotactic prostate cancer radiotherapy with CyberKnife

**DOI:** 10.1002/acm2.70784

**Published:** 2026-09-30

**Authors:** Hilla Magga, Henri Korkalainen, Tuomas Virén, Janne Heikkilä, Jan Seppälä, Akseli Leino

**Affiliations:** ^1^ Department of Technical Physics University of Eastern Finland Kuopio Finland; ^2^ Center of Oncology Kuopio University Hospital Kuopio Finland; ^3^ Department of Nuclear Medicine Satasairaala Hospital Pori Finland

**Keywords:** CyberKnife, deep learning, dose prediction, prostate cancer, radiotherapy

## Abstract

**Background:**

Deep learning‐based (DL) approaches have gained interest in predicting dose distributions in radiotherapy of prostate cancer treated with volumetric modulated arc therapy and intensity‐modulated radiation therapy. Meanwhile, research on predicting dose distributions in high‐precision stereotactic radiotherapy treatments has remained relatively underrepresented.

**Purpose:**

We aimed to expand the previous studies by developing a DL‐based framework for predicting dose distributions for robotic, stereotactic prostate cancer radiotherapy.

**Methods:**

We harnessed a U‐Net‐based convolutional neural network for predicting clinically achievable dose distributions based on CT images, delineated structures, and distance information from the planning target volume. A dataset of 462 patients treated with CyberKnife (Accuray Inc.) utilizing an Iris collimator was divided into training (70%, *n* = 323), validation (10%, *n* = 46), and test (20%, *n* = 93) sets.

**Results:**

In the independent test set, the mean absolute error between the mean doses of predictions and clinical plans was 0.63 Gy for the rectum and 1.04 Gy for the bladder.

**Conclusions:**

The proposed U‐Net‐based model demonstrated the ability to learn and reproduce characteristic dose distributions in CyberKnife prostate cancer radiotherapy. The model may provide patient‐specific dose estimates for setting initial planning objectives to assist in automating treatment planning and improving inter‐planner consistency.

## 1 INTRODUCTION

Prostate cancer is the second most frequent cancer and the fifth leading cause of cancer death among men. In 2022, 1.5 million new prostate cancer cases and 397 000 deaths were estimated worldwide.^1^ Radiotherapy (RT) is one of the most common treatment modalities for prostate cancer. Due to the development of RT techniques^2^, the overall workflow complexity has increased considerably. This increased workload, alongside substantial estimated cancer incidences^3^, causes a remarkable burden on healthcare systems. Thus, there is an urgent need for innovations that automate the RT workflow and reduce clinical workload without compromising treatment quality.

Intensity‐modulated radiotherapy (IMRT) and volumetric modulated arc therapy (VMAT) are often used RT treatment techniques for prostate carcinoma. In these treatments, a total dose of 60 Gy is delivered in 20 fractions conventionally. However, high fraction sensitivity of prostate cancer indicates that larger fraction doses might result in a higher therapeutic benefit.^4^ This has raised interest in studying extreme hypofractionation for prostate cancer (5–10 Gy in 4–7 fractions). However, fewer fractions and larger doses per fraction increase demands for treatment accuracy.

In stereotactic body radiotherapy (SBRT), a high dose of radiation is delivered in a single dose or a few fractions.^5^ One possible treatment modality for prostate SBRT treatments is the CyberKnife (CK) system.^6^ CK is a non‐gantry‐based frameless robotic RT modality^7^ utilizing image guidance during treatment delivery to achieve accurate treatments with steep dose gradients around the target volume.^8^ Multiple noncoplanar and nonisocentric beams are utilized in CK RT treatments^9^, making the treatment planning process complex. During the process, treatment planning parameters are optimized through trial and error without prior knowledge of the optimal treatment plan.

By enabling automated tools, deep learning (DL) and machine learning have substantial potential in healthcare applications, providing insights into large datasets.^10^, ^11^, ^12^ In RT, DL methods hold the potential to assist in treatment planning by making the process more automated, thus increasing consistency and efficiency while delivering optimized and individualized treatment plans.^13^ Most previous studies have developed DL models for IMRT^14^, ^15^, ^16^ and VMAT^17^, ^18^, ^19^ treatments, while research on SBRT treatments remains in the minority.^20^ Only a few studies^20^, ^21^ have developed DL models for predicting dose distributions of CK treatments, for brain and lung cancer, respectively. Compared to VMAT, dose distributions of CK plans differ in target and normal tissues.^6^ Thus, new CK‐specific DL models for dose prediction are needed.

To expand the previous studies, we aimed to develop a DL‐based model to predict achievable dose distributions for CK treatments of prostate cancer. The model was designed to learn CK‐specific dose distribution features while reliably predicting doses to organs at risk (OARs). To achieve this, we utilized a large dataset from CK prostate cancer treatments. Ultimately, predicted dose distributions are intended to support clinical decision‐making and automate the CK treatment planning. To the best of our knowledge, this is the first study predicting dose distributions for CK prostate cancer treatments.

## 2 METHODS

### 2.1 Data

In this study, a dataset of 462 prostate cancer patients treated with CyberKnife VSI (Accuray, USA) at the Kuopio University Hospital (KUH), Kuopio, Finland, during the years 2017–2022 was selected. The patients received a total dose of 35 Gy or 36.25 Gy in five fractions. An Iris collimator was used for treatment delivery. A dose planning computed tomography (CT) scan (Siemens Somatom Definition AS Open 2020 RT Pro edition, Germany) and a magnetic resonance imaging scan (GE Signa Artist 1.5 T, USA) were acquired for each patient at the beginning of the treatment course. On these images, OARs and the prostate were delineated. Margins of the planning target volume (PTV) were 3 mm posterior and 5 mm in all other directions from the delineated prostate. For every patient, only one treatment planning CT was included in the dataset. Overall, 36 patients had a hip implant in the dataset. Treatment plans were created in the Precision treatment planning system with a ray tracing algorithm (Accuray, USA, Software Versions: MultiPlan 4.6.0 and 5.2.1 (before January 2021), Precision 3.1.0.0 and 3.5.0.1 (after January 2021)). Treatment plan optimization was done with the Sequential algorithm during the years 2017–2021, and with the VOLO algorithm during the years 2021–2022. All treatment plans were normalized so that 95% of the PTV received the prescription dose (35 Gy or 36.25 Gy, depending on the clinical decision). Treatment planning objectives are presented in Table 1.

**TABLE 1:** Treatment planning objectives used for CyberKnife prostate cancer treatments at our clinic.

Structure	Limit [Gy]	Volume [cc/%]
Rectum	38	0.05 cc
	36	1 cc
	18.13	50 %
Bladder	38	0.05 cc
Bladder wall	18.3	15 cc
Urethra	40	40 %
Penile bulb	20	0 cc
Femoral head	30	10 cc
Bowel	25	1 cc

For every patient in the dataset, a planning CT, delineated structures, and a clinically approved dose distribution were extracted from the clinical database of the hospital. The Ethics Committee of the Hospital District of Northern Savo, Kuopio, Finland, has granted a favorable statement for the collection and analysis of the data at KUH (78/2021 and 23/2024). In the dataset, the number of slices per patient varied between 345 and 473, and the median number of slices was 401. All images were downsampled from 512 × 512 to 256 × 256 pixels to reduce the computational load. Delineated structure information was used to create patient‐specific structure masks. In a structure mask, voxel values were discretized to correspond to PTV, OARs, body, and outside of the body. Voxels were assigned a value of 1 in the PTV, 2 in the rectum, 3 in the bladder, 4 in the bowel, 5 in the penile bulb, and 6 in the cavernosum. Voxels outside of the body were discretized to −1 and 0 within the body. To address the overlapping areas of the delineated structures in the structure mask, the PTV and OARs were prioritized. The overlapping areas were considered as the structure with the highest priority. The prioritization order from the highest to the lowest was selected as PTV, rectum, bladder, bowel, penile bulb, cavernosum, and body. The structure masks were utilized only for the DL model. For the analyses of the model's performance, full volumes of OARs were considered instead of prioritizing structures in overlapping areas. Two different prescription doses were included in the dataset, as patients received either a total dose of 35 Gy or 36.25 Gy. For this study, we normalized all plans to a dose of 35 Gy by utilizing cumulative dose‐volume histograms (DVHs) with a bin size of 0.1 Gy.

### 2.2 Model architecture

We modified a convolutional neural network U‐Net3+^22^ for a regression problem of predicting clinically achievable dose distributions for CK prostate cancer treatments (Figure 1). The number of filters was 64, 128, 256, and 512 from the top layer to the bottom layer. Thus, we modified the original model architecture by reducing the last layer. For the convolution, we used 3 × 3 kernels. The input of the model had three channels that were 256 × 256 pixels each (Figure 2). The first and second channels were CT and the corresponding structure mask. The third channel was a distance map^23^ where every pixel represented the shortest distance to the edge of the PTV. Distance maps were calculated in 3D for voxels within the body by utilizing the delineated PTV. In 3D distance maps, each voxel within the body represented the shortest distance to the edge of the PTV (in mm). Original distance maps were then scaled approximately between zero and one by dividing all values by 400. Voxels outside of the body were set to −1 and inside the PTV to 0. The chosen scaling results in a similar scale of distance maps among all the patients, which is also on a similar scale to other inputs. This promotes stable model training. Even though scaled distance maps were calculated in 3D, the model uses them slice‐by‐slice. The model's output was a 256 × 256 image representing the predicted dose distribution.

**FIGURE 1:**
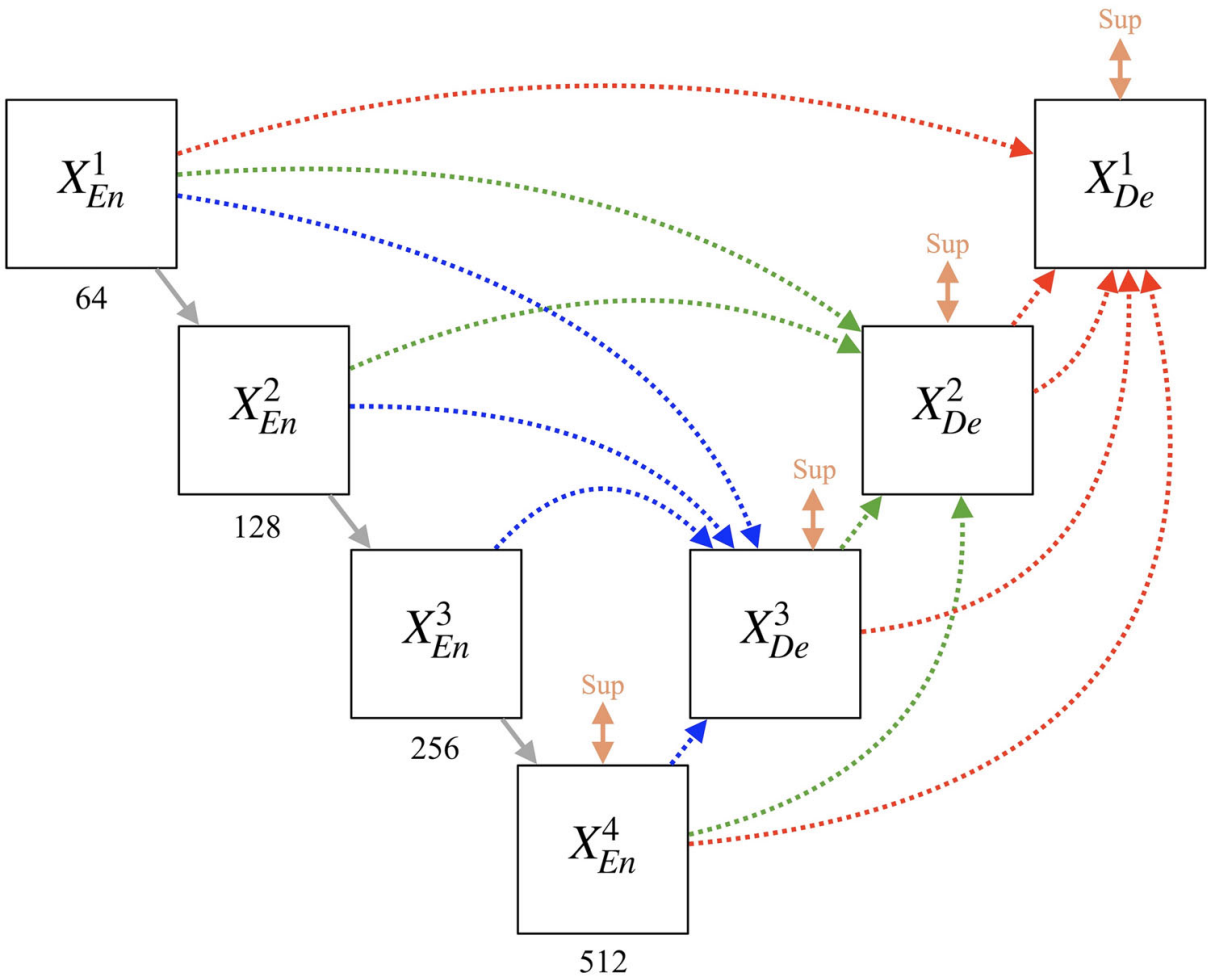
A schematic figure of our model's architecture based on U‐Net3+.^22^ Boxes represent encoder (denoted with lower capital En) and decoder (De) nodes of the convolutional layers of the model. Downsampling between encoder layers is denoted with grey arrows. Dotted arrows represent the full‐scale inter‐skip connections between the encoder and decoder and the full‐scale intra‐skip connections between decoders. The depth of each node is presented under the box. Supervision (Sup) by clinical plans is denoted with orange arrows.

**FIGURE 2:**
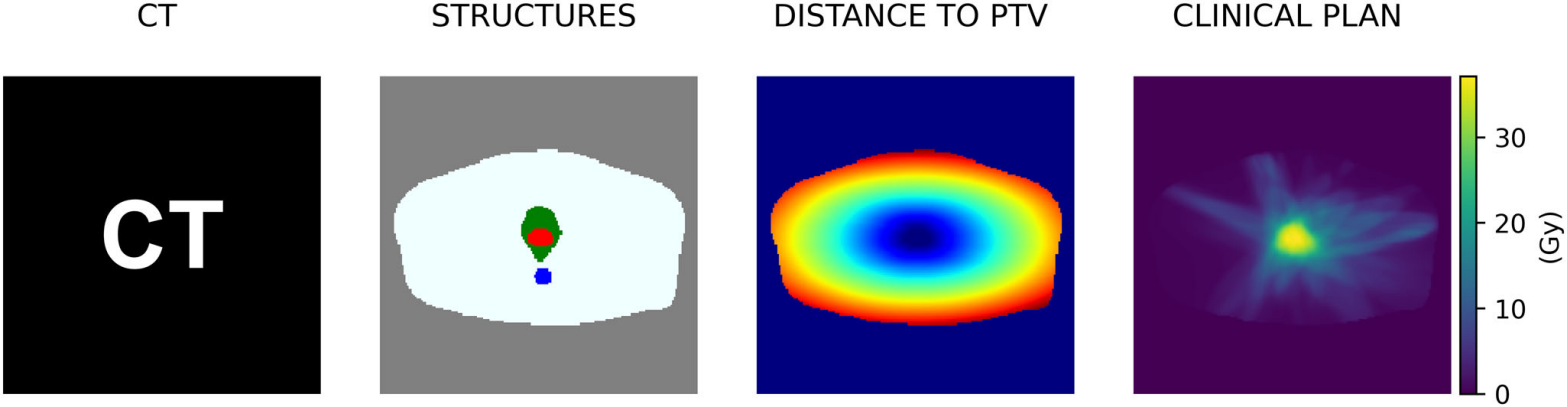
Computed tomography image (CT, omitted for anonymization), delineated structures (red = planning target volume, blue = rectum, green = bladder), and distance information from the planning target volume (PTV) of a patient in the test set. These images were used to predict the dose distribution of the patient.

### 2.3 Model training and evaluation

The dataset of 462 patients was randomly divided into a training, validation, and test set. The model was trained with 70% (*n* = 323) of the patients and evaluated during training with a validation set of 10% (*n* = 46). Patients with hip implants were divided randomly into all three sets. As a result, 25 patients with hip implants were in the train set, three in the validation set, and eight in the test set. During training, the batch size was 15 slices, and the minimized loss function was selected to be mean squared error (MSE). The model was trained for 35 epochs, and the final model was selected based on the smallest MSE of the validation set. The optimization was executed with the Adam optimizer with an initial learning rate of 0.0003. During training, the learning rate was updated with a cosine annealing with warm restarts learning rate scheduler. The gradients were accumulated for 16 batches, after which the model parameters were updated. No data augmentation (e.g., rotation or cropping) was performed on the dataset to preserve the geometric and spatial fidelity of the data. This is essential in radiotherapy, where image geometry is directly linked to beam propagation in the medium. Non‐physical transformations such as scaling can distort path lengths and attenuation properties, leading to physically inconsistent representations. The DL model was created with PyTorch 2.1.0 and the data processing pipeline with TorchIO 0.19.2. Training was executed on a server with an NVIDIA RTX 3090 GPU with 24 GB of VRAM.

The performance of the final model was evaluated with a test set of 20% (*n* = 93) of the patients with respect to the treatment planning objectives of CK prostate cancer treatments at our clinic, presented in Table 1. Doses corresponding to these treatment planning objectives were calculated for clinical plans and predicted dose distributions of the test set and compared with two‐sided Wilcoxon signed‐rank tests. For the rectum and bladder, we presented cumulative DVHs of patients corresponding to the 90th percentile, median, and 10th percentile of absolute error between the structure mean dose in predictions and clinical plans. Bland‐Altman plots were used to visualize the differences between predicted and clinical dose distributions. For further statistical comparison, we performed two‐sided Wilcoxon signed‐rank tests to compare predicted and clinical dose distributions in mean doses of PTV and selected OARs. In addition, we performed two‐sided Wilcoxon signed‐rank tests for doses received by at least a certain percentage (D1%, D5%, D10%, and D50%) of the rectum and bladder in predicted and clinical dose distributions of the test set. The significance level was chosen to be 0.05 for all statistical tests.

## 3 RESULTS

The mean absolute error (MAE) between PTV mean doses in predictions and clinical plans was 0.36 Gy (Table 2). For the selected OARs, MAEs of mean doses varied between 0.50–1.04 Gy (Table 2). For dose metrics corresponding to treatment planning objectives (Table 1), MAEs varied between 0.70–2.74 Gy (Table 3). Predictions slightly underestimated (*p *< 0.05) rectal D_0.05 cc_ and D_1 cc,_ as well as D_10 cc_ of both femoral heads (Table 3). In contrast, the model slightly overestimated (*p *< 0.05) bladder D_0.05 cc_ (Table 3).

**TABLE 2:** Mean absolute error of structure mean doses in clinical plans and DL‐based predictions.

	PTV	Rectum	Bladder	Bowel
Median (1st–3rd quartile) clinical dose [Gy]	37.47 (37.28–37.73)^*^	9.82 (8.21–13.62)	10.50 (9.17–11.90)^*^	1.46 (0.86–3.09)
Median (1st–3rd quartile) predicted dose [Gy]	37.20 (37.11–37.28)^*^	10.08 (8.40–13.27)	10.93 (8.71–12.40)^*^	1.48 (1.00–2.45)
MAE of the means [Gy]	0.36	0.63	1.04	0.50

*Note*: Mean absolute error (MAE) for the mean doses in the planning target volume (PTV) and selected organs at risk (OARs) between clinical plans and DL‐based predictions in the test set. The table also presents medians and 1st and 3rd quartiles of structure mean doses for PTV and OARs in predicted and clinical dose distributions.

* Statistical significance (*p* < 0.05) in a two‐sided Wilcoxon signed‐rank test.

**TABLE 3:** Doses to volumes that correspond to treatment planning objectives.

Structure	Median (1st–3rd quartile) of clinical dose [Gy] in cc/Dx%	Median (1st–3rd quartile) of predicted dose [Gy] in cc/Dx%	MAE [Gy]
Rectum 0.05 cc	38.10 (36.80–39.40)^*^	37.60 (36.90–38.60)^*^	0.77
Rectum 1 cc	36.00 (33.80–38.30)^*^	35.60 (33.60–38.00)^*^	0.78
Rectum D50%	6.20 (5.50–9.00)	6.50 (5.60–8.60)	0.76
Bladder 0.05 cc	37.50 (36.90–38.00)^*^	37.80 (37.50–38.10)^*^	0.70
Bladder wall 15 cc	10.90 (8.30–15.40)	11.50 (8.20–14.80)	1.34
Penile bulb 0 cc	17.17 (14.51–22.61)	18.31 (13.94–22.74)	2.74
Left femoral head 10 cc	10.10 (9.00–11.10)^*^	9.00 (8.20–10.00)^*^	1.18
Right femoral head 10 cc	9.50 (8.30–10.70)^*^	8.80 (8.00–9.90)^*^	0.85
Bowel 1 cc	4.90 (3.30–10.03)	4.80 (2.78–6.85)	1.47

*Note*: Median, 1st and 3rd quartiles of doses to volumes in predicted and clinical dose distributions of the test set. Presented doses to volumes correspond to treatment planning objectives presented in Table 1.

* Statistical significance (*p* < 0.05) in a two‐sided Wilcoxon signed‐rank test.

There were no systematic offsets in mean doses of PTV and OARs between predicted and clinical dose distributions (Figure 3). Statistical comparisons revealed that in predictions, PTV and bladder mean doses slightly differed (*p* < 0.05) from clinical doses (Table 2). Median of PTV dose means was slightly lower for predicted dose distributions (median [interquartile range (IQR)] 37.20 Gy [0.17 Gy]) than for clinical plans (median [IQR] 37.47 Gy [0.45 Gy]) (Table 2). In contrast, the median of bladder dose means was higher in predicted dose distributions (median [IQR] 10.93 Gy [3.69 Gy]) than in clinical plans (median [IQR] 10.50 Gy [2.73 Gy]) (Table 2). Overall, when compared to clinical plans, predicted doses received by PTV were slightly underestimated, and doses received by the bladder were slightly overestimated.

**FIGURE 3:**
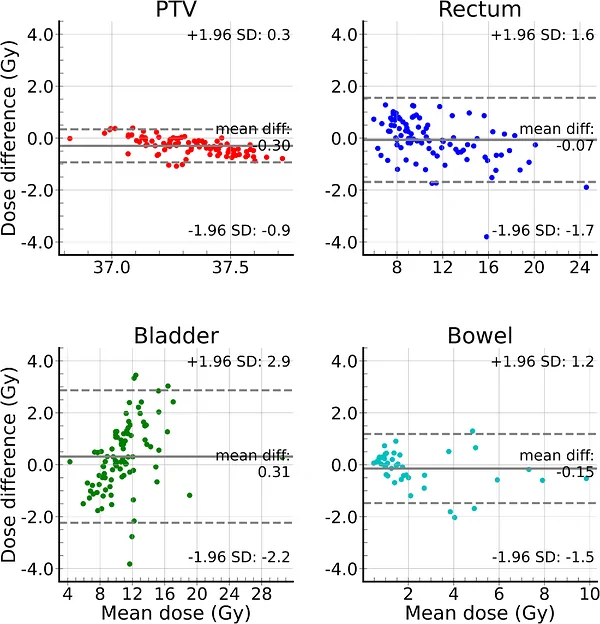
Bland‐Altman plots of the mean doses in clinical plans and DL‐based predictions of the test set for the planning target volume (PTV) and selected organs at risk. The bowel was not delineated for all patients. Dose differences were calculated by subtracting the structure mean doses of clinical plans from the structure mean doses of predictions. SD, standard deviation.

We illustrated DVHs corresponding to the 10th percentile, median, and 90th percentile of the absolute error between structure mean doses in predictions and clinical plans for the rectum (Figure 4) and bladder (Figure 5). For DVHs of PTV, predictions were highly similar to clinical plans in all cases, and the largest differences were observed in the high dose range. Figures S1–S3 illustrate selected axial slices of patients representing the 10th percentile, median, and 90th percentile of the absolute error in rectal mean dose. For the selected axial slices, delineated structures, distance to the planning target volume (PTV), clinical plan dose, predicted dose, and the clinical minus predicted dose difference are presented.

**FIGURE 4:**
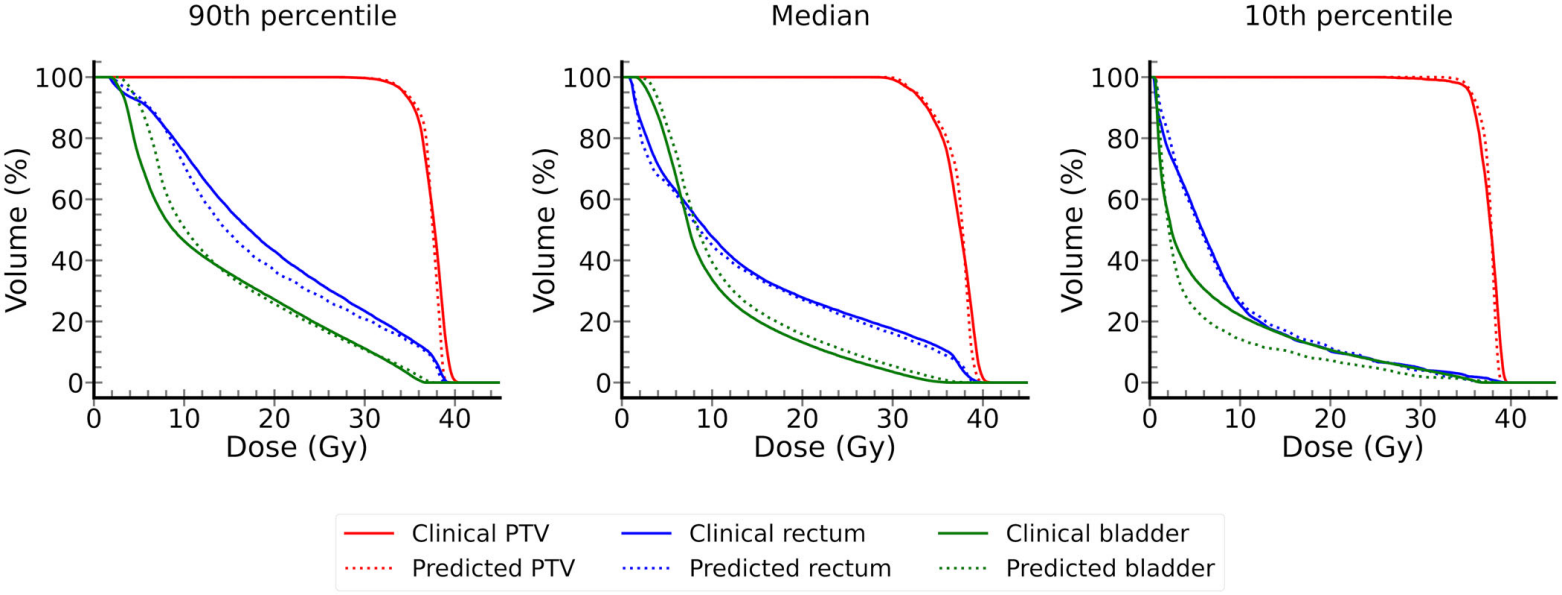
Dose‐volume histograms (DVHs) of clinical and predicted dose distributions in the planning target volume (PTV) and selected organs at risk. DVHs correspond to the 90th percentile, median, and 10th percentile of absolute error between mean doses of the rectum in predictions and clinical plans. In the illustrated figures, the absolute error is largest for the 90th percentile and smallest for the 10th percentile.

**FIGURE 5:**
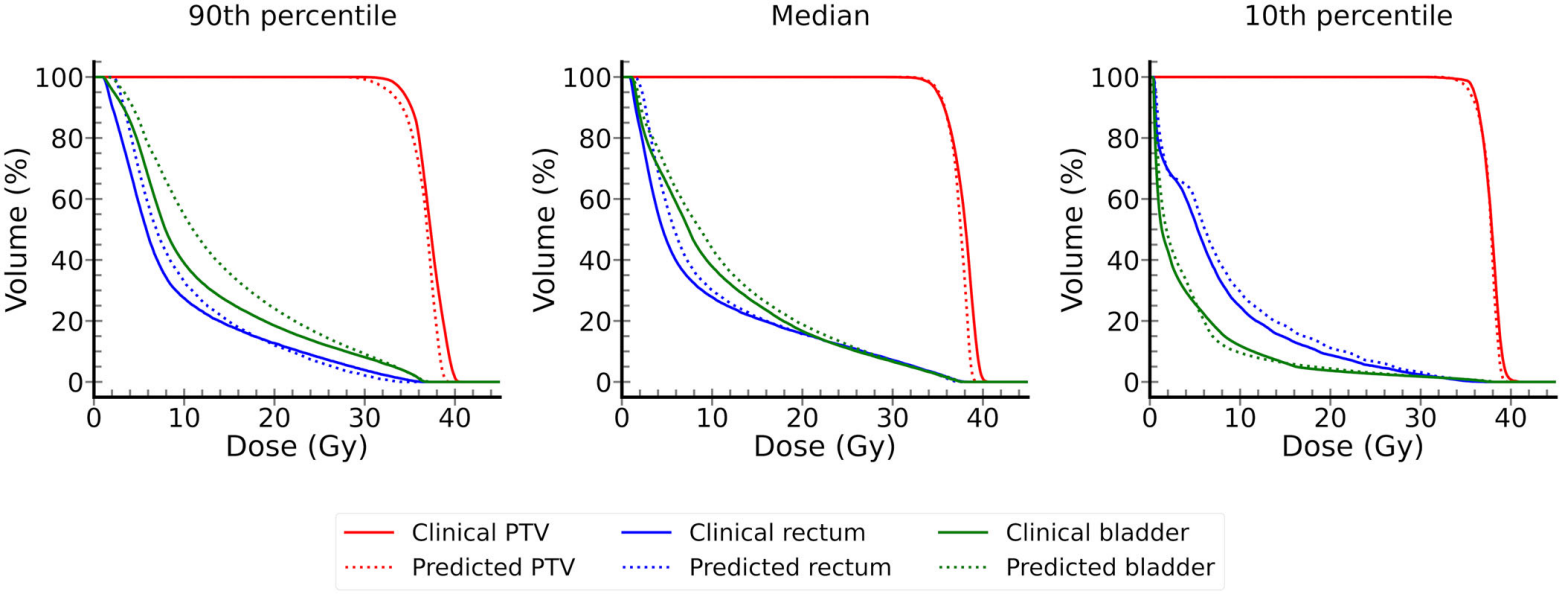
Dose‐volume histograms (DVHs) of clinical and predicted dose distributions in the planning target volume (PTV) and selected organs at risk. DVHs correspond to the 90th percentile, median, and 10th percentile of absolute error in mean doses of the bladder in predictions and clinical plans. In the illustrated figures, the absolute error is largest for the 90th percentile and smallest for the 10th percentile.

For D1%, D5%, D10%, and D50%, MAEs ranged between 0.75–1.14 Gy for the rectum and between 0.59–1.62 Gy for the bladder (Table 4). Bland–Altman plots did not show a consistent offset across all four metrics (Figures 6 and 7). In predicted dose distributions, rectal D1% and D5% were lower than in the corresponding clinical plans. For the bladder, D1% and D50% were higher for predicted dose distributions than for clinical plans (all *p* < 0.05; Table 4).

**TABLE 4:** Mean absolute error of the rectum and bladder in clinical plans and dose predictions.

	D1%	D5%	D10%	D50%
**Rectum**				
Median (1st–3rd quartile) clinical dose [Gy]	36.6 (35.0–38.7)^*^	31.0 (28.0–36.5)^*^	24.7 (19.8–33.1)	6.2 (5.5–9.0)
Median (1st–3rd quartile) predicted dose [Gy]	36.1 (34.7–38.1)^*^	30.4 (27.9–35.6)^*^	24.0 (20.4–30.6)	6.5 (5.6–8.6)
MAE [Gy]	0.75	0.83	1.14	0.76
**Bladder**				
Median (1st–3rd quartile) clinical dose [Gy]	36.1 (35.4–36.8)^*^	32.0 (28.8–34.0)	26.3 (21.4–29.5)	6.8 (6.1–8.0)^*^
Median (1st–3rd quartile) predicted dose [Gy]	36.6 (35.6–36.9)^*^	32.2 (29.0–33.9)	26.2 (21.3–29.6)	7.5 (6.0–9.0)^*^
MAE [Gy]	0.59	1.06	1.62	1.34

*Note*: Mean absolute error (MAE) of Dx% (dose received by at least x% of the volume of the organ at risk) between clinical plans and DL‐based predictions of the test set. The table also presents medians and 1st and 3rd quartiles of the D1%, D5%, D10%, and D50% of rectum and bladder corresponding to clinical plans and DL‐based predictions.

* Statistical significance (*p* < 0.05) in a two‐sided Wilcoxon signed‐rank test.

**FIGURE 6:**
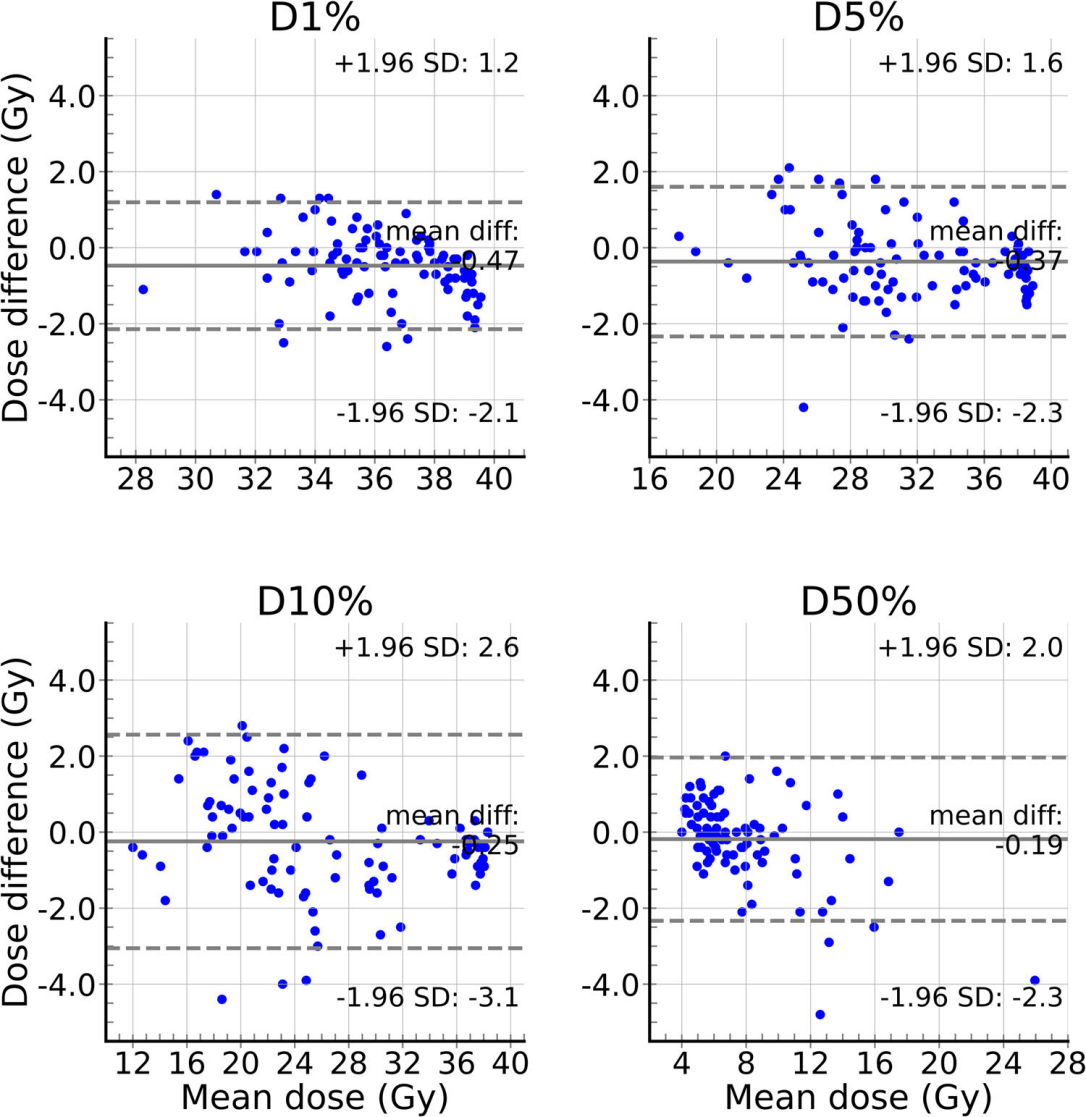
Bland‐Altman plots corresponding to D1%, D5%, D10%, and D50% of the rectum in clinical plans and DL‐based predictions of the test set. Dx% is the dose received by at least x% of the rectal volume. Dose differences were calculated by subtracting the Dx% of clinical plans from the Dx% of predictions. SD, standard deviation.

**FIGURE 7:**
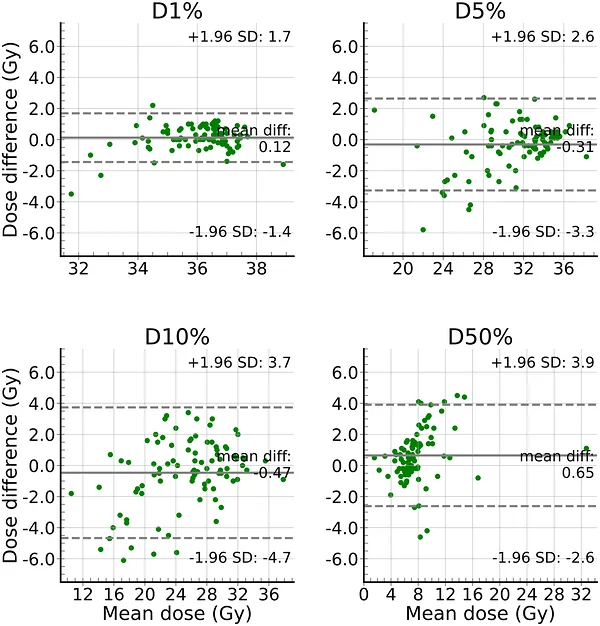
Bland‐Altman plots corresponding to D1%, D5%, D10%, and D50% of the bladder in clinical plans and DL‐based predictions of the test set. Dx% is the dose received by at least x% of the bladder volume. Dose differences were calculated by subtracting the Dx% of clinical plans from the Dx% of predictions. SD, standard deviation.

## 4 DISCUSSION

We developed a DL‐based method for predicting dose distributions slice‐by‐slice for CK prostate cancer treatments from CT images, structure masks, and distance to PTV information by utilizing a large clinical dataset of 462 prostate cancer patients. The developed DL model was able to predict dose distributions that correspond to clinical planning objectives, demonstrating promising clinical potential. MAEs were 0.36 Gy for PTV mean dose and 0.50–1.04 Gy for mean doses of the selected OARs.

The MAEs for the doses of PTV and OARs were generally small, suggesting promising prediction accuracy (Tables 2, 3, 4). Compared to clinical plans, mean doses of PTV were slightly underestimated in predictions (median: clinical 37.47 Gy, predictions 37.20 Gy; *p* < 0.05). The highest doses and mean doses in the bladder were slightly overestimated (*p* < 0.05) in predictions (median: D1% = 36.6 Gy, D50% = 7.5 Gy, D_0.05 cc_ = 37.80 Gy) compared to clinical plans (median: D1% = 36.1 Gy, D50% = 6.8 Gy, D_0.05 cc_ = 37.50 Gy) (Tables 3 and 4). In contrast, rectum doses were slightly underestimated (median: D1% = 36.1 Gy, D5% = 30.4 Gy, D_0.05 cc_ = 37.60 Gy, and D_1 cc_ = 35.60 Gy) compared to clinical plans (median: D1% = 36.6 Gy, D5% = 31.0 Gy, D_0.05 cc_ = 38.10 Gy, and D_1 cc_ = 36.00 Gy). In both femoral heads, D_10 cc_ was slightly underestimated (*p* < 0.05) in the predictions (medians: 9.00 Gy and 8.80 Gy) compared to clinical plans (medians: 10.10 Gy and 9.50 Gy). However, the differences were small and may not result in a clinically meaningful impact.

For dose metrics corresponding to the local planning objectives (Table 1), MAEs ranged from 0.70 to 2.74 Gy (Table 3). These results suggest that the model could provide patient‐specific reference values for setting initial planning objectives before treatment planning system optimization and the traditional trial‐and‐error approach to treatment planning. Such estimates may help guide the optimization process, although their effect on planning efficiency and final plan quality requires prospective evaluation.

Overall, the DL model slightly underestimated the mean dose in PTV, the highest rectal doses, and D_10 cc_ of femoral heads. In addition, the highest doses and the mean dose received by the bladder were slightly overestimated in the predicted dose distributions compared with clinical plans. Several factors may have contributed to these differences. First, the model did not receive explicit information about overlaps between the PTV and OARs, which may limit its ability to reproduce the steep dose gradients and clinical trade‐offs occurring in these regions. Second, downsampling of the images and dose distributions may have smoothed steep dose gradients and introduced partial‐volume effects. This may particularly affect small structures and near structure boundaries, thereby contributing to high‐dose and small‐volume metrics. Finally, the dataset included prescription doses of both 35 Gy and 36.25 Gy, although the same planning constraints (Table 1) were applied clinically to both groups. The higher prescription can produce a steeper dose gradient towards the rectum. Despite all treatment plans being normalized to 35 Gy for the model, the normalization may not have fully preserved these prescription‐specific dose characteristics.

In recent years, DL methods have been widely applied to the RT treatment planning process. In addition to commercially available knowledge‐based treatment planning systems, many studies have utilized convolutional neural networks to predict dose distributions. However, many studies in the literature rely on relatively small datasets of single treatment sites. In addition, most previous studies have developed DL models for conventional linear accelerator treatments, particularly for VMAT^14^, ^15^ and IMRT treatments,^17^, ^18^, ^19^ while research on SBRT has remained in the minority^20^, ^21^. The present study extends these works to prostate CK treatment, where numerous noncoplanar and nonisocentric beam directions create substantially greater delivery complexity and inter‐patient variability. This study evaluates an anatomy‐based model in a large clinical cohort and relates its predictions to dose metrics corresponding to local treatment‐planning objectives. The findings therefore provide evidence that clinically relevant dose metrics from CK plans can be estimated from patient anatomy despite the absence of explicit beam information.

Previous dose‐prediction studies have followed both anatomy‐only and beam‐informed approaches. Conditioning a model on a predefined beam configuration may improve configuration‐specific spatial accuracy, particularly in low‐ and intermediate‐dose regions. However, this approach requires a patient‐specific candidate beam configuration to be defined before dose prediction, either through manual selection or by obtaining an initial configuration from the treatment planning system.^24^ Nonetheless, both options require manual work from the planner, and the resulting prediction is therefore conditional on the selected configuration. While beam‐informed models have provided promising results, the high level of freedom in CK treatments challenges the planner to find patient‐specific, clinically optimal beam angles that can be used as an input. In contrast, an anatomy‐only model can provide an earlier estimate before beam selection and treatment planning system optimization.

Nevertheless, beam‐informed prediction remains a promising pathway for improving aspects of the dose distribution that are not fully determined by anatomy. In the present study, gamma passing rates were markedly lower in the low‐dose region than in the high‐dose region (Table S1), indicating reduced spatial agreement where dose deposition is more strongly influenced by the selected beam paths. Although these gamma results do not directly determine the clinical usefulness of predictions, they suggest that the anatomy‐only model has difficulty reproducing configuration‐dependent low‐ and intermediate‐dose patterns. Incorporating representations of candidate beam directions or beam paths could help the model associate delivery geometry with these spatial dose features. Future studies should directly compare anatomy‐only and beam‐informed models in the same CK cohort. Such comparisons should evaluate not only prediction accuracy and final plan quality, but also planning time, manual intervention, and overall workflow complexity, as the need to generate and review a candidate beam configuration may reduce the practical benefit of beam‐informed prediction. A hybrid workflow may offer another pathway: an anatomy‐only model could first provide patient‐specific estimates for the initial planning objectives. Then, the treatment planning system could generate a preliminary beam configuration. For this configuration, a second, beam‐informed model could refine dose estimates or optimization objectives. Future work should determine whether anatomy‐only, beam‐informed or sequential use of both approaches provides the greatest practical assistance during CK treatment planning.

In addition to the comparison of anatomy‐only and beam‐informed models, future research is warranted to further improve the prediction accuracy and translate the predictions into deliverable treatment plans. One potential pathway to improve prediction accuracy would be changing slice‐by‐slice anatomical information to 3D^25^ and utilizing a structure‐focused loss function^26^, which may improve the representation of inter‐slice anatomy, structure boundaries, and steep dose gradients. Furthermore, one potential approach would be to implement physics‐informed neural networks that incorporate knowledge of radiation transport and dose deposition, which have provided promising results in IMRT dose predictions.^27^

Moreover, the current prediction model represents CK treatments delivered with the Iris collimator. In contrast, newer CK platforms are also equipped with multi‐leaf collimators (MLC). The developed DL model could be utilized to represent dose distributions achieved with an Iris collimator, serving as a valuable tool when transferring to MLC‐based planning. Ultimately, it could be used to estimate the difference between an Iris plan (predicted) and an MLC plan (manually planned). In addition, a similar model could further be trained on a dataset of prostate cancer patients treated with the MLC. Together, these two DL models would provide a novel tool for comparing patient‐specific effects of collimator choice in CK prostate cancer treatments.

The quality, quantity, and diversity of the training data will remain central to the performance and generalizability of these models. Planning objectives, optimization strategies, and accepted clinical trade‐offs may vary between institutions and planners. Thus, a model trained on local historical plans may reproduce center‐specific practice and treatment plans might be planner‐dependent within the same treatment center.^21^ Therefore, collaborating multi‐institutionally and creating open‐access datasets would be crucial in the field. Ultimately, dose prediction DL models could help in improving and standardizing the RT treatments across treatment centers.

This study has some limitations that need to be considered. Firstly, the developed DL model predicts dose distributions instead of deliverable treatment plans. Our dose predictions work as a starting point for treatment planning, easing the optimization process and reducing manual parameter tuning. Further research is needed to study the possibilities of DL‐based dose predictions in automating the treatment planning process, as well as the added clinical value of the predicted dose distributions. For example, utilizing beam information and other standard inputs of the treatment planning process as inputs for the DL model would be essential to study, as these could further support automating the treatment planning process. Secondly, our dataset contained two different prescription dose levels. To address this, all clinical doses were normalized by utilizing cumulative DVH curves. However, the prescription dose affects dose distribution, especially in the rectum and bladder high‐dose regions. Thus, the dose distributions of the two prescription doses might have characteristic features regardless of the normalization. In addition, 36 patients had hip implants, but delineated hip implants were not included in the structure information input of the DL model. Thus, information about the patient's hip implant is not directly introduced to the DL model. This might negatively affect the prediction accuracy of the DL model on patients with hip implants and lead to non‐feasible dose distributions in which beams penetrate the hip implant.

Moreover, we only utilized a 2D prediction model incorporating distance maps from the PTV and did not systematically compare different model architectures or 3D approaches after preliminary testing. Despite having the distance maps, the model may overlook some inter‐slice correlations that are not present in the PTV distance. A systematic comparison of different approaches was outside the scope of this study, and further research is needed to determine the effects of different approaches on prediction accuracy. Finally, even though the results of this study were promising, further research is still needed to generalize them to different institutions and treatment sites. Instead of deliverable treatment plans, the predicted dose distributions are supposed to serve as a starting point for treatment planning. Further studies on the clinical feasibility of the predicted dose distributions are needed but outside the scope of this study. This was a single‐center study with a reasonably large dataset of 462 patients. For validating the results and extending the research, an international multi‐center collaboration would be essential.

## 5 CONCLUSIONS

In this study, we developed a DL model for predicting dose distributions for CK prostate cancer treatments from patient anatomy and distance‐to‐PTV information. The model demonstrated promising prediction accuracy and holds the potential for supporting and streamlining the treatment planning process. Predictions could provide insights into achievable dose distributions, ultimately reducing manual parameter tuning during the treatment planning process. By serving as an initial starting point for the treatment planning process, predicted dose distributions could improve the consistency of the treatment planning task. Further research and multi‐center collaboration are needed for investigating the conversion of predictions into deliverable plans, evaluating the achieved workflow benefit, and extending the approach to other treatment sites and collimator types.

## AUTHOR CONTRIBUTIONS

Henri Korkalainen, Tuomas Virén, Janne Heikkilä, Jan Seppälä, and Akseli Leino devised the work and the main conceptual ideas. Tuomas Virén prepared the data that Hilla Magga preprocessed for the analyses. Hilla Magga, Henri Korkalainen, and Akseli Leino carried out analyses and interpretation of the data. Hilla Magga visualized the results and drafted the manuscript. All authors have revised the manuscript critically, approved the submitted version for publication, and agreed to be accountable for all aspects of the work.

## CONFLICT OF INTEREST STATEMENT

The authors declare no conflicts of interest.

## ETHICS STATEMENT

The Ethics Committee of the Hospital District of Northern Savo, Kuopio, Finland, has granted a favorable statement for the collection and analysis of the data at KUH (78/2021 and 23/2024).

## Supporting information



Supporting Information

## Data Availability

Authors are not able to share data at this time.
