# Supplementary material for: Deep learning‐based dose prediction for stereotactic prostate cancer radiotherapy with CyberKnife
Source: J Appl Clin Med Phys. 2026 Sep 30;27(10):e70784. doi: 10.1002/acm2.70784 (PMC13626863; doi:10.1002/acm2.70784)
Supplement: Supplementary file 1 [file ACM2-27-e70784-s001.docx]

**Supplementary material for: Deep learning-based dose prediction for stereotactic prostate cancer radiotherapy with CyberKnife**

# *Supplementary material*

# Methods

The performance of the final model was evaluated with a test set of 20% (*n* = 93) patients. To assess combined dosimetric and spatial agreement between clinical plans and predicted dose, we calculated global and local 2D and 3D gamma passing rates between the clinical plans and predicted dose distributions. Global gamma passing rates were normalized to the maximum dose in each patient’s clinical plan. In contrast, local gamma passing rates were normalized to the reference dose in the center pixel/voxel of the considered area/volume in the patient’s clinical plan. The gamma passing rates were calculated using a 3 %/3 mm criterion separately for the high dose region (>50% dose of the clinical plan maximum dose), for the low dose region (10–50% dose), and for the cutoff region (10–100% dose). The gamma passing rates were calculated separately for the whole test set and for patients with a hip implant (*n* = 8).

**Results**

Median 3D gamma passing rates varied between 0.41–0.90 in global analysis and between 0.21–0.86 in local analysis (Supplementary table 1). In 2D gamma analyses, median 2D gamma passing rates varied between 0.34–0.80 in global analysis and between 0.12–0.71 in local analysis (Supplementary table 1). Supplementary figures 1-3 show clinical dose, predicted dose, and difference maps for test set patients representing the 90th percentile, median, and 10th percentile, respectively, of the absolute error in rectal mean dose.

Supplementary table 1: Gamma passing rates for predicted dose distributions calculated with a 3 %/3 mm criterion.

|  | 2D | 3D |
| --- | --- | --- |
| Local gamma |  |  |
| High dose region |  |  |
| Test set | 0.71 (0.67–0.76) | 0.86 (0.82–0.89) |
| Implants of test set | 0.66 (0.61–0.70) | 0.82 (0.77–0.85) |
| Low dose region |  |  |
| Test set | 0.16 (0.13–0.18) | 0.26 (0.22–0.29) |
| Implants of test set | 0.12 (0.11–0.15) | 0.21 (0.19–0.24) |
| Cut off 10% of max. dose |  |  |
| Test set | 0.19 (0.16–0.21) | 0.29 (0.25–0.32) |
| Implants of test set | 0.15 (0.14–0.18) | 0.24 (0.23–0.28) |
|  |  |  |
| Global gamma |  |  |
| High dose region |  |  |
| Test set | 0.80 (0.75–0.83) | 0.90 (0.87–0.93) |
| Implants of test set | 0.75 (0.71–0.80) | 0.87 (0.83–0.90) |
| Low dose region |  |  |
| Test set | 0.42 (0.38–0.45) | 0.49 (0.44–0.52) |
| Implants of test set | 0.34 (0.30–0.40) | 0.41 (0.36–0.46) |
| Cut off 10% of max. dose |  |  |
| Test set | 0.44 (0.40–0.47) | 0.51 (0.46–0.54) |
| Implants of test set | 0.36 (0.32–0.42) | 0.43 (0.39–0.49) |

Median, 1st and 3rd quartiles of local and global gamma passing rates in the test set (*n* = 93) and for hip implant patients (*n* = 8) in the test set. The gamma passing rates were calculated separately for the high dose region (>50% dose of the clinical plan maximum dose), for the low dose region (10–50% dose), and for the cutoff region (10–100% dose).

Supplementary figure 1: Selected axial slices showing delineated structures (red = planning target volume, blue = rectum, green = bladder), distance to the planning target volume (PTV), clinical plan dose, predicted dose, and the clinical minus predicted dose difference. The patient represents the 90th percentile of the absolute error in rectal mean dose. From 401 CT slices, slices 180–215 are shown at 5 mm intervals.

Supplementary figure 2: Selected axial slices showing delineated structures (red = planning target volume, blue = rectum, green = bladder, turquoise = bowel), distance to the planning target volume (PTV), clinical plan dose, predicted dose, and the clinical minus predicted dose difference. The patient represents the median of the absolute error in rectal mean dose. From 401 CT slices, slices 180–215 are shown at 5 mm intervals.

Supplementary figure 3: Selected axial slices showing delineated structures (red = planning target volume, blue = rectum, green = bladder, yellow = cavernosum), distance to the planning target volume (PTV), clinical plan dose, predicted dose, and the clinical minus predicted dose difference. The patient represents the 10th percentile of the absolute error in rectal mean dose. From 401 CT slices, slices 180–215 are shown at 5 mm intervals.
